# Effects of a commercial product containing guaraná on psychological well-being, anxiety and mood: a single-blind, placebo-controlled study in healthy subjects

**DOI:** 10.1186/1477-5751-12-9

**Published:** 2013-05-25

**Authors:** Gianluca Ivan Silvestrini, Franca Marino, Marco Cosentino

**Affiliations:** 1 Center for Research in Medical Pharmacology, University of Insubria, Via Ottorino Rossi n. 9, Varese, VA 21100, Italy

**Keywords:** Guaranà, *Paulinia cupana*, Labelled instructions, Psychological well-being, Anxiety, Mood, Humans

## Abstract

**Background:**

Guaranà (*Paulinia cupana*) seed extracts are increasingly popular worldwide for their stimulant, cognitive and behavioral effects. To assess the effects on psychological well-being, anxiety and mood of a commercially available guaranà preparation taken regularly over several days according to the labelled dosages and instructions, 27 healthy volunteers were enrolled in a prospective, randomized, single-blind, placebo-controlled, crossover study.

**Results:**

Guaranà 350 mg × 3 daily just after breakfast or placebo were given for 5 consecutive days. Assessment was performed one day after the last intake and included the psychological well-being (PWB) scales, the self-rating anxiety state scale (SAS), and the Bond–Lader mood scales. There were no significant differences between guaranà and placebo in any of the 6 areas of PWB, in SAS, as well as in any of the 16 mood scales.

**Conclusions:**

In healthy subjects a 5-day treatment with a commercial preparation of guaranà used according to labelled instructions provided no evidence for any major effects on psychological well-being, anxiety and mood. Considering the increasing popularity of guaranà-containing products sold as dietary supplements for fitness purposes, controlled studies are strongly warranted to assess their benefits in comparison to the labelled claims.

## Background

Guaranà (*Paulinia cupana*) is an Amazonian creeping shrub which has been used for centuries by Amerindian tribes for several indications, including: cardiovascular drug, preventive for arteriosclerosis, pain-reliever, astringent, stimulant, tonic, to treat diarrhea, hypertension, fever, migraine, neuralgia and dysentery [1]. Guaranà seed extracts however over the last few decades became increasingly popular worldwide mainly for their stimulant and thermogenic actions, as ingredients in many herbal formulas, energy drinks, and protein bars [1].

Several studies addressed the stimulant, cognitive and behavioral effects of guaranà [2]. In particular, various double-blind, placebo-controlled studies in human subjects showed that guaranà acutely improves some parameters of cognitive performance without effects on mood [3], enhances secondary memory performance and increases alert and some content mood ratings [4], increases speed and accuracy of performing rapid visual information processing tasks and attenuates mental fatigue associated with extended task performance [5]. At least one controlled study however failed to report any effects on psychomotor speed and accuracy, memory, visuomotor performance, planning and problem solving [6]. Conflicting results have been reported also in the few studies which addressed the effects of guaranà after long-term administration. Indeed, in two recent randomised, cross-over, placebo-controlled studies in breast cancer patients, guarana was effective in reducing fatigue during systemic chemotherapy [7] but had no effect on radiation-induced fatigue and depression [8]. Moreover, in a double-blind study it was reported that long-term administration of guaranà had no significant effects on the cognition of normal, elderly volunteers [9].

Commercial products currently available on the market usually contain guaraná extracts which are concentrated and standardized to the caffeine content. Commonly recommended dosages correspond to 20–30 mg of caffeine per day while usually no instructions are provided about optimal duration of use. Indeed, guaranà is generally regarded as safe by the Food and Drug Administration Center for Food Safety and Applied Nutrition [10]. These products are usually marketed with claims of effectiveness, but the weight of the evidence is very small for most of them. A recent survey found that guaranà is among the most common herbal ingredients sold over the Internet even for use as legal alternatives to illicit drugs of abuse [11]. Additional concern comes from the observation that potential uses suggested in websites include mood enhancement as well as treatment of depression, together with the suggestion that guaranà may increase feelings of well-being. To our best knowledge however no evidence exists so far which might support those claims, except for a report showing, after acute administration of guaranà, increased alert ratings and improved content ratings measured by use of the Bond-Lader mood scales [4]. Guaranà contains large amounts of caffeine, as well as trace amounts of theophylline and theobromine, which are believed to contribute significantly to guaranà effects [1]. Indeed moderate intake of caffeine has been associated with less depressive symptoms, although its effects on mood never received thorough investigation [12].

Considering the increasing popularity of guaranà-containing products and the paucity of evidence regarding their actual effectiveness, we decided to assess in healthy subjects the effects on psychological well-being, anxiety and mood of a commercially available guaranà preparation used according to the labeled dosages and instructions.

## Results

### Subjects

Twenty-seven volunteers (9 female and 18 male, age (mean ± SD) 25.4 ± 6.4 years) were enrolled in a prospective, randomized, single-blind, placebo-controlled, crossover protocol evaluating the effects on psychological well-being, anxiety and mood of the intake for five consecutive days of a guaranà extract at 360 mg × 3 daily just after breakfast, according to labelled advice All the subjects enrolled successfully concluded the study, regularly taking all the capsules in the two treatment periods and participating to all the 4 evaluation sessions. No subjects reported any adverse event in any of the evaluation sessions.

### Psychological well-being

There were no significant differences among evaluation sessions in the scores of subjects taken as a whole in any of the 6 areas of psychological well-being, and in particular there was no significant difference between the effects of guaranà and placebo (Table 1).

**Table 1:** Scores from the PWB scales for the six areas of psychological well-being

**Areas of psychological well-being**	**Pretreatment**	**Placebo**	**Washout**	**Guaranà**	**Two-way ANOVA (F, DFd, P)**	**Guaranà vs Placebo (P)**
**Autonomy**	64.5 ± 9.0	65.4 ± 10.4	64.9 ± 9.2	64.7 ± 10.9	0.267, 62.30, 0.805	0.921
**Environmental mastery**	60.8 ± 9.1	61.4 ± 10.1	62.6 ± 8.9	61.9 ± 10.1	1.207, 63.40, 0.311	0.965
**Personal growth**	66.9 ± 6.3	66.3 ± 8.1	66.0 ± 7.9	66.3 ± 7.8	0.382, 59.33, 0.711	1.000
**Positive relations**	64.4 ± 7.6	63.3 ± 8.4	62.7 ± 8.4	62.5 ± 7.9	1.538, 59.08, 0.221	0.781
**Purpose in life**	63.6 ± 9.2	64.4 ± 8.2	64.7 ± 8.8	63.6 ± 8.8	0.722, 53.84, 0.495	0.810
**Self-acceptance**	61.4 ± 13.1	61.7 ± 11.1	62.4 ± 10.9	62.8 ± 11.4	0.928, 57.63, 0.410	0.540

Data are means ± SD.

When subjects were divided according to gender, no signficant difference was observed in males (guaranà vs placebo: autonomy, P = 0.727; environmental mastery: P = 0.979; personal growth: P = 1.000; positive relations: P = 0.698; purpose in life: P = 0.298; self-acceptance: P = 0.991), while in females after guaranà there was a significant increase in the self-acceptance area (59.1 ± 9.3 with placebo vs 63.0 ± 9.4 with guaranà, P = 0.033) but not in any of the other areas (autonomy, P = 0.955; environmental mastery: P = 0.626; personal growth: P = 0.999; positive relations: P = 1.000; purpose in life: P = 0.329).

Subjects were also divided in two groups according to the median score obtained for each area after placebo, to test for any difference between lower- and higher-scoring subjects, however no significant difference occurred between the effects of guaranà and placebo in either groups (lower-scoring group: autonomy, P = 0.990; environmental mastery: P = 0.769; personal growth: P = 0.934; positive relations: P = 0.989; purpose in life: P = 0.998; self-acceptance: P = 0.412; higher-scoring group: autonomy, P = 0.339; environmental mastery: P = 0.959; personal growth: P = 0.784; positive relations: P = 0.065; purpose in life: P = 0.453; self-acceptance: P = 0.994).

### Self-rating anxiety

The scores obtained in the evaluations sessions were: pretreatment 33.8 ± 7.0, placebo 32.4 ± 6.9, washout 31.2 ± 6.7, guaranà 32.2 ± 7.3.

There were no significant differences among evaluation sessions in the scores of subjects taken as a whole (F = 2.410, DFd = 57.49, P = 0.093; guaranà vs placebo: P = 0.992).

The effect of guaranà was not significantly different from the effect of placebo even in comparisons according to gender (female: P = 0.059; male: P = 0.895) and to the low or high scores obtained after placebo (low scores: P = 0.909; high scores: P = 0.797).

### Mood

There was no significant effect of the treatments on mood as assessed by the Bond–Lader mood scales (Table 2).

**Table 2:** Scores from the Bond–Lader VAS

**Mood factors**	**Pretreatment**	**Placebo**	**Washout**	**Guaranà**	**Two-way ANOVA (F, DFd, P)**	**Guaranà vs Placebo (P)**
**Alert**	29.9 ± 14.9	33.1 ± 18.3	28.7 ± 13.8	30.0 ± 15.1	0.948, 59.95, 0.404	0.733
**Calm**	40.0 ± 22.0	39.3 ± 23.6	39.0 ± 21.7	38.0 ± 22.1	0.099, 55.15, 0.915	0.989
**Content**	26.4 ± 16.4	33.7 ± 21.9	29.8 ± 17.6	30.4 ± 14.8	1.671, 64.43, 0.190	0.858

Data are means ± SD.

The effect of guaranà and placebo did not significantly differ even according to gender (female: alert, P = 0.362; calm: P = 0.999; content: P = 0.971; male: alert, P = 0.998; calm: P = 0.979; content: P = 0.901) or to the lower or higher scores obtained after placebo (lower-scoring group: alert, P = 0.190; calm: P = 0.533; content: P = 0.565; higher-scoring group: alert, P = 0.065; calm: P = 0.810; content: P = 0.395).

### Statistical power analysis

The least hypothetical differences between the effects of guaranà and placebo which could be detected in the whole study population in each test at the 0.05 significance level and with a power value of 0.80 were as follows:

– PWB scales (areas of psychological well being):

• Autonomy: 2.80

• Environmental mastery: 2.43

• Personal growth: 2.10

• Positive relations: 2.32

• Purpose in life: 2.32

• Self-acceptance: 2.32

– SAS scale: 2.21

– Bond–Lader VAS:

• Alert: 8.57

• Calm: 11.48

• Content: 11.59

## Discussion

The purpose of our study was to assess in a population of healthy subjects the effects of a commercially available guaranà preparation on psychological well-being, anxiety and mood when the product was used according to the labelled dosages and instructions. Results provide no evidence for any major effects of guaranà on the psychological tests employed. The only significant difference in favour of guaranà was found in female subjects and it was limited to just one of the six areas of psychological well-being (namely, self-acceptance), thus it cannot be excluded that it represents just a random finding. Given the null results reported, it is important to consider whether the study was adequately powered to detect significant effects if they existed. Power analysis of the results show that the least hypothetical differences between the effects of guaranà and placebo which could be detected in our study were in the range 2.1-2.8 for PWB scales and 2.2 for the SAS scale, while they were in the range 8.6-11.6 for the Bond–Lader scales. Least hypothetical differences are therefore very small for the PWB scales and for the SA scale. In particular, each of the PWB scales may range 14–84 [13-15], thus the least detectable differences would be just 3.5% of the entire interval, which is highly unlikely to be somehow clinically significant. For the SAS scale total scores range from 20–80 and clinically relevant intervals are: 20–44 normal, 45–59 mild to moderate anxiety, 60–74 marked to severe anxiety, 75–80 extreme anxiety [16]. Intervals are therefore nearly seven-fold higher than the least detectable differences in the present study, thus it is unlikely that any significant effects could have been overlooked. As for the Bond Lader scales, least detectable differences in our study were in the range 8.6-11.6. The Bond Lader scales were used in at least one previous study showing positive results with guaranà [4]: after acute administration of guaranà to 26 healthy subjects (18 females, 8 males) there were statistically significant increases of alert and content ratings, the size being in both cases on average around 6–7 units, a difference which could have been overlooked in our setting. Nonetheless, according to our results the average differences observed between placebo and guaranà in either alert, content and calm were in the range 1.3-3.3, therefore unlikely to be clinically relevant and in any case half or less those found in the study by Haskell et al. [4]. It should be noted that also Kennedy et al. [3] examined the effects of a single dose of guaranà extract on mood using the Bond-Lader scales. They reported no signifcant effects, however they also provided no figures and it is therefore not possible to compare those results with the present findings.

Studies investigating the neurobehavioural effects of guaranà in humans so far considered mainly the acute effects. Kennedy and co-workers [3] used 75 mg of a dried ethanolic extract of guaranà and found positive effects on some tasks of the Cognitive Drug Research (CDR) computerised assessment and on the Serial subtraction task, and in particular improvements to speed of attention, secondary memory, serial subtractions and speed of sentence verification, but no effects on the Bond-Lader mood scales. The same group in a subsequent study [5] showed that 222 mg of guaranà (containing 40 mg caffeine) added to vitamin/mineral effervescent tablets improved task performance, in comparison to placebo, in terms of both increased speed and accuracy of performing the Rapid Visual Information Processing and attenuated mental fatigue associated with extended task performance. Haskell and co-workers [4] used a standardized guaranà extract containing 11-12% caffeine and found positive effects on secondary memory performance in the CDR computerised assessment with 37.5 and 75 mg, on alert ratings of the Bond-Lader mood scales with 300 mg and on content ratings with 37.5, 75, 150 and 300 mg. Fernandes Galduróz and de Araújo Carlini [6], who failed to report any effects with guaranà, evaluated 1000 mg guaranà containing 21 mg caffeine on tests of psychomotor speed and accuracy (letter cancellation), working memory (digit span and digit symbol substitution), memory (free recall and learned material) and the Mosaic test (involving visuomotor performance, planning and problem solving). No effect was also found on anxiety and quality of sleep.

Just a few studies addressed the effects of guaranà after prolonged administration. The study by de Oliveira Campos and co-workers [7] examined the effects of 50 mg guaranà by mouth twice daily for 21 days in 75 breast cancer patients receiving systemic chemotherapy using a double-blinded procedure and found positive results on several fatigue scales with no worsening of sleep quality or anxiety or depression. In another double-blinded, cross-over study, da Costa Miranda et al. [8] randomized 36 patients with breast cancer undergoing adjuvant radiation therapy to either guaranà 75 mg daily for 28 days or to placebo and found no effect on radiation-induced fatigue and depression. Fernandes Galduróz and de Araújo Carlini [9] administered 1 g of powdered guaranà (containing 2.1% caffeine) per day for five months to normal, elderly volunteers who were evaluated at baseline and after 3 and 5 months. Evaluations included the Mini-Mental State as screening for dementia, the Digital Span to assess immediate memory, the Free Recall for recent memory, Digital Symbol for psychomotor activity and concentration, Cancellation tests for vigilance and attention, the Mosaic test for visual and spatial organization, the Rave-Progressive Matrices for general abilities, and in addition quality of sleep and anxiety were assessed. No significant effects were detected on any of the functions tested and in addition no subjects reported any rise in libido or improved sexual performance.

Based upon available literature, it appears therefore that guaranà has been investigated mainly as a stimulant, in agreement with its chemical composition, while the possible effects of guaranà on mood have been only occasionally considered. So far only Haskell et al. [4] reported some positive results on mood, while Kennedy et al. [3] failed to observe any significant effects. Nonetheless, in both cases single doses of guaranà were studied and short-term effects were reported.

Guaranà-containing products are usually recommended for regular use: this is the main reason why our study was concerned with the effects of guaranà regularly taken according to the labeled dosages and instruction over several days. The results of our study, which found no effect of guaranà on psychological well-being, anxiety and mood, are in line with previous studies reporting no activity of guaranà [6,9]. We decided to assess the effects of guaranà on psychological well-being since the possible increase of “feelings of well-being” is among the most common claims for guaranà-containing products in particular on the Internet, where guaranà is one of the most popular herbal ingredients sought for the purpose of increasing alertness and fitness [11], however to our knowledge no scientific studies existed so far which addressed this issue. In our study we used a guaranà extract commercially available and standardized to 2.5% caffeine. Enrolled subjects took a total of 1080 mg/day, according to labelled commercial instructions and corresponding to 27 mg caffeine per day. Likely explanations for lack of effects and apparent discrepancies with other studies (e.g. Haskell and co-workers [4] who found positive effects on some ratings of the Bond-Lader mood scales) include the possibility that the selected tasks were insensitive to the levels of treatment used, or the testing regimens were not adequate to observe the effects of guaranà. Indeed, caffeine is one of the main chemicals in guaranà seeds, its content being usually 4-8% [1]. The half-life of caffeine however is about 5 h (see e.g. [17]), therefore any acute effects are likely to disappear in a short time, while the supposed long-term benefits of frequent caffeine consumption (e.g. reduced risk of type 2 diabetes and liver cancer) possibly require years of more or less regular intake (see e.g. [18,19]): it cannot be excluded that the same applies to the possible effects of caffeine on mood, which so far never received thorough investigation [12].

In conclusion, the present study failed to observe any significant effect of a 5-day treatment with a commercial preparation of guaranà on psychological well-being, anxiety and mood in healthy subjects. A number of adverse events are reported with the use of herbal food supplements containing guaranà, including irritability, heart palpitations, anxiety and other central nervous system events [20], especially in children, adolescents, and young adults with seizures, diabetes, cardiac abnormalities, or mood and behavioral disorders or those who take certain medications [21]. Also in view of the weak evidence available regarding the actual effectiveness of commercial products containing guaranà, caution shoud be always used and advised to potential users. Considering the increasing popularity of products containing guaranà – as well as a number of other traditional herbs – and sold as dietary supplements for health and fitness purposes, controlled studies are strongly warranted to assess their benefits in comparison to the labelled claims.

## Methods

### Subjects

We enrolled 27 volunteers (9 female and 18 male, age (mean ± SD) 25.4 ± 6.4 years). Subjects taking part in the study were acquaintances of the students attending the Course in Motorial Sciences at the Faculty of Medicine, University of Insubria (Varese, I). All the subjects reported good health, were not cigarette smokers and did not report use of over-the-counter, medication (including hormonal contraceptive methods) and/or illicit drugs in the previous three months. Participants were told that the study aimed at the evaluation of the effects of mood of a commerncial product containing guaranà used according to the commercial advice and that, to this end, they would have received during distinct 5-day periods in random sequence capsules containing guaranà or corn starch. All the participants provided written informed consent and the study was performed according to ethical guidelines for research on humans.

### Study products

We used a commercial guaranà extract kindly provided by Anderson Research (Cassino, FR, Italy; http://www.anderson-research.com). The extract contained caffeine 2.5% (w/w) and was prepared in 360 mg capsules. According to labelled instructions, 3 capsules/day should be taken, with no recommendations regarding duration of use. Placebo capsules contained 360 mg of cornstarch.

### Study design

The study was a prospective, randomized, single-blind, placebo-controlled, crossover study evaluating the effects on psychological well-being, anxiety and mood of the intake for five consecutive days of a guaranà extract at 360 mg × 3 daily just after breakfast, according to labelled advice.

### Procedure

Each participant was required to attend a total of 4 evaluation sessions (pretreatment, treatment 1, washout, treatment 2), which comprised completion of the psychological well-being scales (PWB) [13], the self-rating anxiety state scale (SAS) [16], and the Bond–Lader Visual Analogue Scales [22]. Testing took place during the morning between 9:00am and 11:00am in a room of the laboratories, and subjects were visually isolated from each other during the whole session. The pretreatment session (day 1) was identical to the others and was devised to eliminate practice effects and to familiarize participants with the evaluation procedures. Participants were then randomized to guaranà or placebo and received 15 capsules and the instructions about how to take them each day (3 capsules/day immediately after breakfast with half glass of water) for five consecutive days (days 2–6). Then participants underwent the second evaluation session (day 7, treatment 1) and then a washout period of additional five days (days 8–12), which was followed by an evaluation session (day 13, washout) and by a second 5-day period of treatment (days 14–18, crossover: placebo or guaranà according to what they received in the first period). The day after the end of the second treatment period (day 19) participants underwent the final evaluation session (treatment 2). Evaluation sessions following treatment periods always included a standard interview about any possible adverse events.

Subjects were blind to treatments. Random allocation to guaranà or placebo was accomplished by use of a random number calculator (QuickCalcs, GraphPad Software, La Jolla California USA, http://www.graphpad.com). During the first period of treatment 9 subjects received guaranà and 15 received placebo, and were subsequently switched to placebo and guaranà, respectively, during the second period. Before each period of treatment, each subject received 15 capsules (either guaranà or placebo, 3 per day for 5 consecutive days) and was asked to bring back any remaining capsules on the day of the evaluation, to check for compliance. Capsules were handed together with the brochure of the commercial product containing guaranà, to expose subjects to standard information provided when the product is normally purchased.

During the study, subjects were asked to abstain from alcoholic and caffeine-containing beverages, except for a maximum of 4 cups of coffee (italian “espresso”) per day, and to abstain also from coffee in the morning of the weekly testing session. No other dietary restrictions were imposed.

### Psychological well-being (PWB) scales

The long form (84 questions) of the PWB scales developed by Carol D. Ryff [13,14] was used. The scales consist of series of statements reflecting the six areas of psychological well-being: autonomy, environmental mastery, personal growth, positive relations with others, purpose in life, and self-acceptance. Respondents rate statements on a scale of 1 to 6, with 1 indicating strong disagreement and 6 indicating strong agreement. Responses are totaled for each of the six categories (about half of the responses are reverse scored). For each category, a high score indicates that the respondent masters that area in life, while a low score shows that the respondent struggles to feel comfortable with that particular concept. PWB scales have been recently validated for use in the italian population [15].

### Self-rating anxiety scale (SAS)

The SAS [16] was used to quantify the level of anxiety. The scale is a 20-item self-report assessment device including measures of state and trait anxiety. Respondent is asked to indicate how much each statement applies to her or him. Each question is scored on a Likert-type scale of 1–4 (based on the following replies: “a little of the time,” “some of the time,” “good part of the time,” and “most of the time”). Overall assessment is done by total score, after reverse scoring some of the responses. The total scores range from 20–80: 20–44 normal, 45–59 mild to moderate anxiety, 60–74 marked to severe anxiety, 75–80 extreme anxiety.

### Bond–lader visual analogue scales (VAS)

The Bond–Lader VAS [22], consisting of sixteen 100-mm visual analogue scales anchored by antonyms (e.g. Alert–Drowsy, Lethargic–Energetic, etc.), were used to assess subjective mood. The scales reflect three key mood factors: “alert” (alert-drowsy, attentive-dreamy, lethargic-energetic, muzzy-clearheaded, coordinated-clumsy, mentally slow-quick witted, strong-feeble, interested-bored, incompetent-proficient), “calm” (calm-excited, tense-relaxed) and “content” (contented-discontented, troubled-tranquil, happy-sad, antagonistic-friendly, withdrawn-sociable). Scores for each item represent the number of mm from the negative antonym. Item scores were summed and averaged to create total scores for each respective factor. Factor scores had a potential range between 0 and 100, with 100 representing highly alert, calm or content. A pen-and-paper version was used and participants were asked to mark each line between the antonyms indicating how they felt at the present time.

### Data analysis

Data are presented as means ± standard deviation (SD) with n indicating the number of observations. Statistical significance of the differences between sessions were evaluated by means of One-Way ANOVA for repeated measures with Tukey’s correction for multiple comparisons. Statistical significance of the differences was also assessed according to gender and to the median score obtained for each scale after placebo, to test for any difference between lower- and higher-scoring subjects.

Statistical analysis was performed using GraphPad Prism version 6.00 for Mac (GraphPad Software). Sample size calculations could not be performed in advance, since there is no general consensus about the smallest average differences which would be scientifically important for each of the scales employed. The power of the study to detect the least hypothetical difference between the effects of guaranà and placebo was therefore determined for each test after completion of the study. Calculations were performed by use of GraphPad StatMate for Windows (GraphPad Software).

## Competing interests

The authors declared that they have not competing interests.

## Authors’ contributions

MC and GIS conceived and designed the study, GIS collected the data, MC, FM and GIS analysed and interpreted results. All the Authors were involved in drafting and revising the manuscript, and gave final approval.
